# Setting conservation priorities in multi-actor systems

**DOI:** 10.1093/biosci/biad046

**Published:** 2023-07-19

**Authors:** Christopher J O'Bryan, Jonathan R Rhodes, Olusegun O Osunkoya, Geoff Lundie-Jenkins, Nisansala Abeysinghe Mudiyanselage, Travis Sydes, Moya Calvert, Eve McDonald-Madden, Michael Bode

**Affiliations:** School of Earth and Environmental Sciences and the Centre for Biodiversity and Conservation Science, University of Queensland, Brisbane, Queensland, Australia; School of Earth and Environmental Sciences and the Centre for Biodiversity and Conservation Science, University of Queensland, Brisbane, Queensland, Australia; Invasive Plant and Animal Science Unit, Department of Agriculture and Fisheries, Biosecurity Queensland, Brisbane, Queensland, Australia; Wildlife and Threatened Species Operations, Department of Environment and Science, Queensland Parks and Wildlife, Toowoomba, Queensland, Australia; School of Earth and Environmental Sciences and the Centre for Biodiversity and Conservation Science, University of Queensland, Brisbane, Queensland, Australia; Far North Queensland Regional Organisation of Councils, Cairns, Queensland, Australia; Invasive Plant and Animal Science Unit, Department of Agriculture and Fisheries, Biosecurity Queensland, Brisbane, Queensland, Australia; School of Earth and Environmental Sciences and the Centre for Biodiversity and Conservation Science, University of Queensland, Brisbane, Queensland, Australia; School of Mathematical Sciences, Queensland University of Technology, Brisbane, Queensland, Australia

**Keywords:** collective action, collaboration, common-pool resource, conservation prioritization, conservation planning, cooperation

## Abstract

Nature conservation is underresourced, requiring managers to prioritize where, when, and how to spend limited funds. Prioritization methods identify the subset of actions that provide the most benefit to an actor's objective. However, spending decisions by conservation actors are often misaligned with their objectives. Although this misalignment is frequently attributed to poor choices by the actors, we argue that it can also be a byproduct of working alongside other organizations. Using strategic analyses of multi-actor systems in conservation, we show how interactions among multiple conservation actors can create misalignment between the spending and objectives of individual actors and why current uncoordinated prioritizations lead to fewer conservation objectives achieved for individual actors. We draw three conclusions from our results. First, that misalignment is an unsuitable metric for evaluating spending, because it may be necessary to achieve actors’ objectives. Second, that current prioritization methods cannot identify optimal decisions (as they purport to do), because they do not incorporate other actors’ decisions. Third, that practical steps can be taken to move actors in the direction of coordination and thereby better achieve their conservation objectives.

Prioritization methods are considered the best practice in biodiversity conservation and management resource allocation (Schwartz et al. 2018, Sinclair et al. 2018). These methods are used by government and nongovernment actors globally because they can improve the cost-effectiveness of decisions made with limited resources, are transparent and repeatable, reduce bias, and allow for *post hoc* evaluation and learning (Halpern et al. 2006, Schwartz et al. 2018, Sinclair et al. 2018, Armsworth et al. 2020, Lawler et al. 2020). Recent applications of prioritization tools include the allocation of resources for spatial conservation planning (Kukkala and Moilanen 2013), among listed species on the US Endangered Species Act (Gerber et al. 2018), threatened species in New Zealand (Joseph et al. 2009), management actions for Australian and Canadian wildlife (Brazill-Boast et al. 2018, Carwardine et al. 2019, Walsh et al. 2020), and invasive species eradication on islands (Baker and Bode 2021).

It is reasonable to expect that the process of prioritization will direct resources toward those actions that deliver the largest expected return on investment, typically defined by a combination of the benefit of an action to the stakeholder or decision-maker, the cost of undertaking protective or remedial action, the magnitude of the threat being averted, and the probability of the action being successful (Murdoch et al. 2007, Joseph et al. 2009, Martin et al. 2018). In *ex ante* models of conservation systems, prioritization tools suggest resource allocations that are strongly correlated with an organization's objectives—the conservation features that matter to an organization (figure 1). In contrast, *ex post* analyses of real-world spending often reveal a misalignment between spending and objectives (Halpern et al. 2006, Knight et al.^ 2006^, Tisdell and Nantha 2007, Weiss et al. 2021). For example, in the 1990s, most federal spending on threatened species in the United States of America was being directed to only 10 of the 554 listed taxa. However, these prioritized species faced fewer threats and were less biologically unique than species that did not receive substantial funding (Metrick and Weitzman 1996).

**Figure 1.**
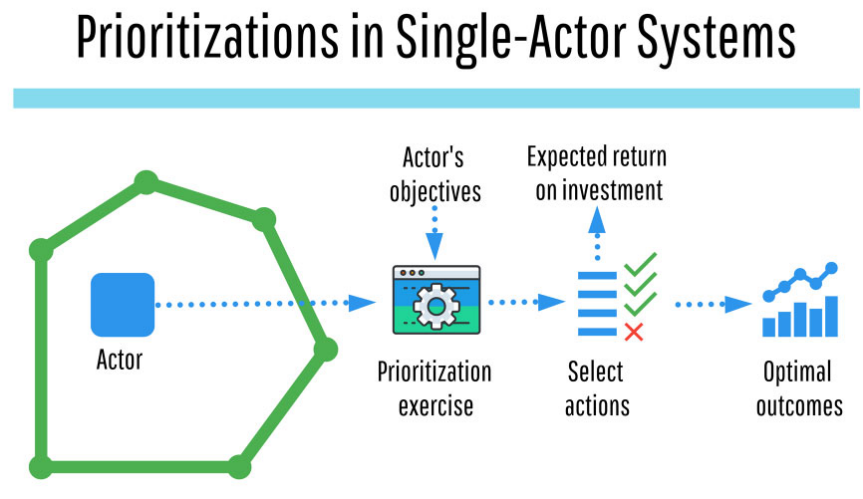
Prioritizations in a single-actor system (the outer polygon). In this system, prioritization decisions are driven by the actor's objectives and by the cost efficiencies produced by the prioritization, which result in strong alignment between their resource allocation and objectives.

Multiple explanations have been offered for observed divergences between conservation objectives and spending. Advocates for conservation prioritization methods often argue that a lack of decision-support tools leads to inefficiency (e.g., Gerber et al. 2018). Proponents of revealed preference theory argue that spending patterns are, in fact, optimal and that the critiques misrepresent the true value systems of decision-makers (e.g., Metrick and Weitzman 1998). Public policy economists observe that conservation features are pure public goods or mixed goods, which inhibits market mechanisms from delivering efficient resource allocation (Tisdell and Nantha 2007). Some social scientists argue that an implementation gap between scientists and on-ground actors hinders the adoption of priority plans (Knight et al. 2006, 2008).

While acknowledging the importance of these factors, we argue for another plausible explanation of the observed misalignment between an actor's (e.g., an organization’s) objectives and their spending (*multi*-*actor systems*). We describe a multi-actor system as the presence of multiple actors who pursue divergent or partially overlapping objectives and who each have access to at least some independent sources of funding (Bodin and Crona 2009, Berardo and Scholz 2010, Bode et al. 2011, Newell et al. 2012). In multi-actor conservation systems, actors tend to pursue overlapping objectives. Their actions can have positive effects on each other's objectives but few negative effects. For example, the habitat protection actions of a bird conservation organization can also benefit threatened plants. This is in contrast to multi-actor systems more broadly, where one actor's decision can adversely affect other actor's objectives—for example, more extensive agriculture can undermine the goals of species conservation organizations (Bode et al. 2011, Gordon et al. 2013, Sayer et al. 2013, Lubell and Morrison 2021). Instances of multi-actor conservation systems include landscape and seascape mosaics in federated nations (Morrison 2017), the not-for-profit land trust sector (Armsworth et al. 2012), and decentralized community-based natural resource management groups such as local forestry (Bixler 2014) and fisheries groups (Berkes 2006, Wilen et al. 2012, Costello et al. 2015, Gelcich et al. 2019).

Multi-actor systems make interactions among actors inevitable, and they give each actor the opportunity to behave strategically—that is, to design their actions in anticipation of or in response to decisions made by other actors (e.g., Bode et al. 2011). Researchers in the fields of environmental governance and economics are aware of these interactions and appreciate their impact on alignment between the spending and objectives of a single actor (e.g., via advocacy coalitions; Silvia 2018, Lubell and Morrison 2021). However, the field of conservation prioritization pays scant attention to this phenomenon. For example, decision support tools such as that of Marxan and Zonation account for multiple objectives (Moilanen et al. 2005, Ball et al. 2009) but do not consider the presence and decisions of any other actors in the system.

In this article, we explore the multi-actor nature of conservation systems and its effect on prioritization. We describe the variety of interaction behaviors commonly observed among conservation actors and explain why these interactions may explain the misalignment between objectives and spending. We then use game-theory models to assess how multi-actor interactions would affect the suitability and performance of existing conservation prioritization tools. Finally, we discuss practical approaches for incorporating these multi-actor interactions into prioritizations, with the aim of improving conservation outcomes without the need to conduct a multi-actor analysis.

## Interaction behavior and hierarchies in multi-actor conservation systems

**Figure 2.**
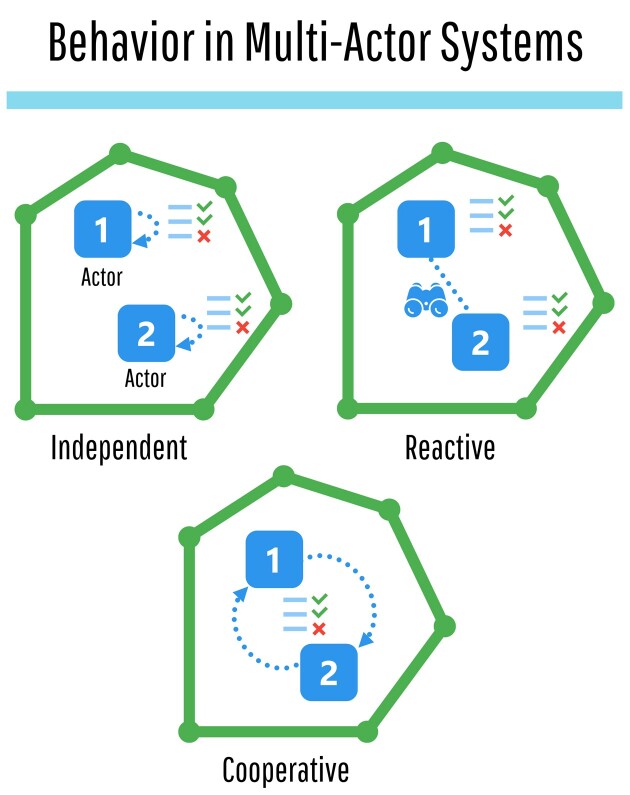
Behavior of conservation actors (the numbered boxes) in multi-actor conservation systems (the outer polygons) consist of independent behavior, reactive behavior, and cooperative behavior. Independent, the conservation actors do not recognize or anticipate the decisions of other actors in the system (top left); reactive, the conservation actors recognize and strategically react to anticipated decisions of other actors (top right) but do not cooperate; cooperative, conservation actors undergo cooperative behavior with other actors in the system (bottom) and not only recognize each other's decisions but actively collaborate to achieve mutually beneficial outcomes.

Within multi-actor conservation systems, actors may commonly exhibit one of three interaction behaviors when prioritizing their actions: They can ignore other actors (i.e., act independently), they can react in response to other actors (i.e., act reactively), or they can seek out cooperation with each other (i.e., act cooperatively; figure 2; Bode et al. 2011). An example of independent action can be found in small conservation nongovernmental organizations that lack the resources to recognize and coordinate with other actors (Guo and Acar 2005, Albers et al. 2008), and this is more likely to occur as the density of actors increases (Margerum 1999, Koch 2011). An example of reactive behavior in conservation systems can be found in the United States, where the conservation actions of government agencies changed the behavior of nearby private land trusts (Albers et al. 2008). The government's actions in this case effectively crowded out investment by the land trusts (Albers et al. 2008). In another example in California's Sierra Nevada Mountains, one rancher's unwillingness to control the invasive yellow star thistle (*Centaurea solstitialis*) reduced nearby ranchers’ incentives to take beneficial action, by increasing their control costs. The result was reduced resource allocation for star thistle management across all ranchers, despite widespread appreciation of its high priority (Epanchin-Niell et al. 2010).

Examples of effective cooperative planning with multiple actors can be found in Australia with the War on Western Weeds (WoWW) in Queensland. The WoWW was a 5-year collaboration funded by the Queensland government to battle the spread of the invasive prickly acacia (*Vachellia farnesiana*) and bellyache bush (*Jatropha gossypiifolia*) and was focused on a community of practice that coordinated the actions of industry, government, natural resource management groups, and scores of individual private landholders (March et al. 2017). The result was increased resource allocation and enhanced capacity for land managers to achieve practical and cost-effective outcomes for both invasive species. Another example of cooperative planning across multiple actors are landscape conservation cooperatives (LCCs) in the United States. LCCs were established by the Department of the Interior in 2010 to drive collaborative conservation planning at the regional scale and have been instrumental in facilitating collaboration across multiple actors ranging from state and federal governments to tribes and First Nations, nongovernmental organizations, and other public and private actors (Baldwin et al. 2018).

**Figure 3.**
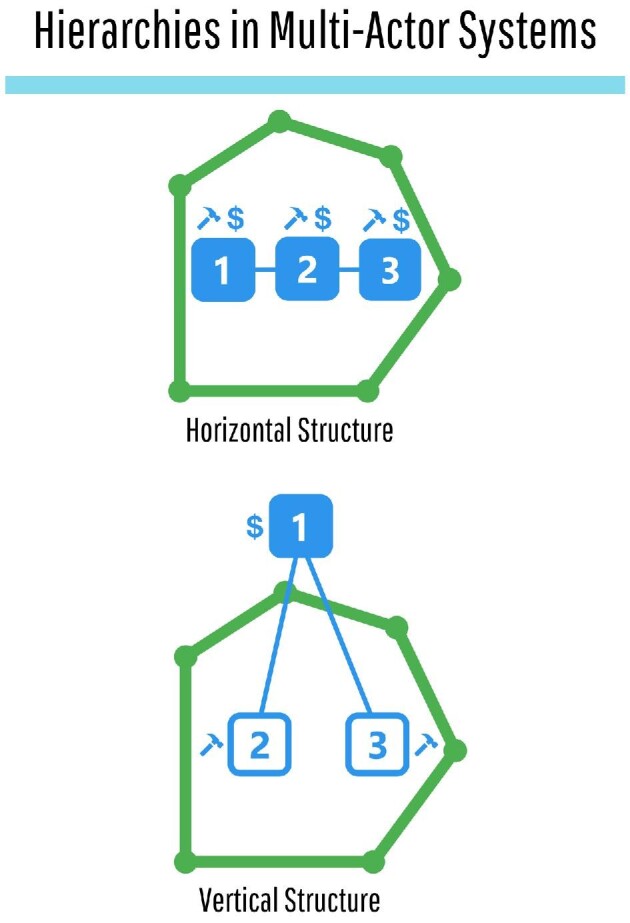
Multi-actor conservation systems (the outer polygons) are organized by horizonal (top) and vertical (bottom) structure. Horizontally organized systems contain multiple on-ground actors that operate alongside each other with their own resources (represented by dollar symbols) and actions (represented by hammer symbols). Vertically organized systems contain funding actors (at the top of the hierarchy; the solid box) that outsource actions to on-ground actors (at the bottom of the hierarchy; the open boxes).

Multi-actor conservation systems may also involve some hierarchical structure, defined by both horizontal and vertical relationships (figure 3; Berkes 2007, Epanchin-Niell et al. 2010, Iacona et al. 2016, Gelcich et al. 2019). In a multi-actor system with vertical structure, actors positioned at the top of the hierarchy can influence the actions and capacity of on-ground actors, who sit lower in the hierarchy, using tools that include funding disbursement and regulatory control (figure 3; Hudson and Bielefeld 1997, Iacona et al. 2016). Vertical structures are generally more common in conservation (Bode et al. 2011, Iacona et al. 2016) and in governance systems, where it is known as principal–agent structure (Ross 1973). Lower-level conservation agents retain some autonomy of action, but this may be anticipated and preempted by the principal actor. Problems with principal–agent relationships arise when there is a conflict in priorities between a principal and an agent (Abbott et al. 2020)—for example, in the case of World Heritage conservation (Morrison et al. 2020). Conversely, horizontal structure involves multiple on-ground actors that operate alongside each other but do not receive funding from each other (figure 3; Albers et al. 2008, Bode et al. 2011, Armsworth et al. 2012). Both dimensions of structure can coexist in the same conservation system. An example of top-down regulatory control and funding disbursement can be found in the state of Queensland, Australia, where the Department of Agriculture and Fisheries exhibits funding and regulatory compulsion on affected and affecting stakeholders (i.e., private landholders, natural resource management groups, and agricultural industries) to manage the spread of pests, diseases, or contaminants in the state (State of Queensland 2014).

## Modeling the consequences of multi-actor structure on conservation actions

To investigate the consequences of multi-actor structure on conservation outcomes and effective conservation resource use for individual actors, we create a theoretical model of a random landscape that contains many different conservation features. These features could be individual taxa (e.g., a particular threatened species), different groups of taxa (e.g., amphibians or primates), or different types of ecosystems (e.g., wetlands or woodlands). Within this landscape, multiple actors expend resources to protect these features, to which they assign different relative value.

Our modeling framework assumes rational behavior to predict how multi-actor governance affects conservation outcomes, in two different ways. First, we measure the degree of alignment between actor objectives and resource allocations, and how this alignment changes with the number of actors and the structure of the sector. That is, are the objectives of the different actors reflected in their spending? Second, we measure how conservation outcomes for individual actors vary among different sectoral structures. Essentially, we asked whether there are certain kinds of multi-actor structure detrimental to achieving conservation outcomes for individual actors.

### Types of conservation actors

We model the conservation sector as *R* rational actors, who seek to protect the same set of *F* conservation features in a single system. These actors all approach resource allocation as a prioritization problem, using the types of prioritization methods commonly available in the conservation literature.

Some are on-ground actors, who undertake conservation actions themselves. On-ground actors are arranged horizontally. Each actor $r\ $has an independent conservation budget ${B}_r$, which they allocate to maximize their conservation goals. We do not consider how these funds are raised, but we do assume that each actor's fundraising outcomes are independent of the other actors, and that they are not affected by their conservation achievements (i.e., there is no feedback between an actor's conservation actions and its fundraising success).

Sitting above the on-ground actors, creating vertical structure, are conservation funders. These actors do not undertake conservation actions themselves and, instead, raise funding, which they distribute to on-ground actor to spend on particular conservation actions (Iacona et al. 2016). We again assume that the resources of different funders are independent of each other and independent of their achievements.

### Conservation actions and outcomes

The actions of each actor, $r,\ $are expressed as a vector of spending decisions ${{\boldsymbol{\beta }}}_{\boldsymbol{r}}$, whose elements ${[ {{{\boldsymbol{\beta }}}_{\boldsymbol{r}}} ]}_f$ denote the proportion of the total budget ${B}_r$ that is allocated to the conservation of feature $f \in \{ {1, \ldots ,F} \}$. The conservation status of a given feature improves when spending is allocated to it, and we assume that this rate of improvement diminishes as the total spending on that feature increases (i.e., there are diminishing marginal returns). Each actor derives utility from the conservation of each feature, proportional to the value ${\alpha }_{rf}\ $that actor $r\ $places on that feature *f*. The total utility flowing to each actor, ${U}_r$, by the actions of all actors across all of the features accrues additively:


(1) \begin{equation*}{U}_r = \mathop \sum \limits_f {\alpha }_{rf}{\left( {\frac{{\mathop \sum \nolimits_r {{\left[ {{{\boldsymbol{\beta }}}_r} \right]}}_f}}{{{c}_f}}} \right)}^z.\end{equation*}

The average cost of undertaking conservation actions for each feature is denoted ${c}_f$. The parameter $0 < z \le 1$ defines the shape of the diminishing marginal returns function. Note that the utility to a particular actor *r* is determined by the allocations of all the actors (i.e., $\mathop \sum _r {\beta }_{rf}$). That is, an actor derives equal utility from a unit of funding allocated to feature *f*, regardless of who it was allocated by.

### Predicting spending decisions when horizontal actors behave independently

If an actor is unaware that other actors are acting in the landscape or has no information about how they are acting, then they will prioritize their spending without considering the actions of the other actor. This is essentially the logic underpinning all existing prioritization tools. An actor *r* allocates their budget ${\beta }_r$ to maximize equation 2:


(2) \begin{equation*}\mathop {\max }\limits_{\left\{ {{\beta }_r} \right\}} \mathop \sum \limits_f {\alpha }_{rf}{\left( {\frac{{{\beta }_{rf}}}{{{c}_f}}} \right)}^z.\end{equation*}

However, their utility will still be calculated accounting for the expenditure of all the actors combined (i.e., following equation 1).

### Predicting spending decisions when horizontal actors behave cooperatively

If actors choose to behave cooperatively, then we assume that they will choose their spending allocation to maximize a joint utility function, which treats the objectives of each actor as equally important. This scenario is equivalent to the actors all pooling their resources and using a standard prioritization tool to determine spending. That is, the cooperative actors will choose to maximize equation 3:


(3) \begin{equation*}\mathop {\max }\limits_{\left\{ {{\beta }_1, \ldots ,{\beta }_R} \right\}} \mathop \sum \limits_r \mathop \sum \limits_f {\alpha }_{rf}{\left( {\frac{{\mathop \sum \nolimits_r {{\left[ {{\beta }_r} \right]}}_f}}{{{c}_f}}} \right)}^z.\end{equation*}

Once again, the utility for each actor will still be calculated accounting to equation 1.

### Predicting spending decisions when horizontal actors behave reactively

When the conservation actors are aware of each other but choose not to formally cooperate, we assume that they act as rational utility maximizers and seek out a Nash equilibrium (in which each actor achieves the desired outcome by not deviating from their initial strategy). This is a joint set of allocation decisions—one for each actor—where each actor is simultaneously at a local maxima with respect to their own decision. That is, if any individual actor altered their allocation unilaterally, their utility would decrease.

We search for the Nash equilibrium using an iterative gradient-based search algorithm. Each actor starts with a random allocation vector. We then allow each actor in turn to vary their allocation vector by a small amount and accept all changes that increase that actor's utility, even if it reduces the utility of another actor or reduces the sum total utility across all actors (see the supplemental material for an example iterative solution of a Nash equilibrium). This method is not guaranteed to identify a Nash equilibrium, but one was found in each of our examples. A complicated multi-actor scenario may also contain more than one Nash equilibrium, but because our utility functions were concave, we found that repeated applications of the iterative method from different initial allocation vectors returned the same solution.

### Predicting spending decisions when vertical actors behave reactively

When the conservation sector has vertical structure, two actors make their choices in a sequence rather than in parallel. Stackelberg games are a logical description of these types of interactions (e.g., Winands et al. 2013) and describe an oligopoly market model of noncooperative strategic game where the leader moves first and others decides how much to move afterward. Stackelberg games include a leader (the funding actor in our case) and a follower (the on-ground actor). Going first confers an advantage on the leader, because they will be able to anticipate the decision of a rational follower, whose decision is defined by the leader's choice (for more details, see von Stackelberg 2011). The goal of the funding actor is to maximize equation 4:


(4) \begin{equation*}\mathop {\max }\limits_{\left\{ {{\beta }_{1f}} \right\}} \mathop \sum \limits_f {\alpha }_{1f}{\left( {\frac{{{\beta }_{1f} + {\beta }_{2f}}}{{{c}_f}}} \right)}^z,\end{equation*}

but in this equation, ${\beta }_{2f}$ is subject to the choices of the on-ground actor who is maximizing equation 5:


(5) \begin{equation*}\mathop {\max }\limits_{\left\{ {{\beta }_{2f}} \right\}} \mathop \sum \limits_f {\alpha }_{2f}{\left( {\frac{{{\beta }_{1f} + {\beta }_{2f}}}{{{c}_f}}} \right)}^z.\end{equation*}

Note that the difference between these utility functions is in the value coefficients (the ${\alpha }_{if}$ values). We solve for the Nash equilibrium of this Stackelberg game by exhaustively searching through all actions available to the leader, where the actions of a rational follower are conditional on the leader's action.

## Misalignment occurs between objectives and spending in multi-actor conservation systems

**Figure 4.**
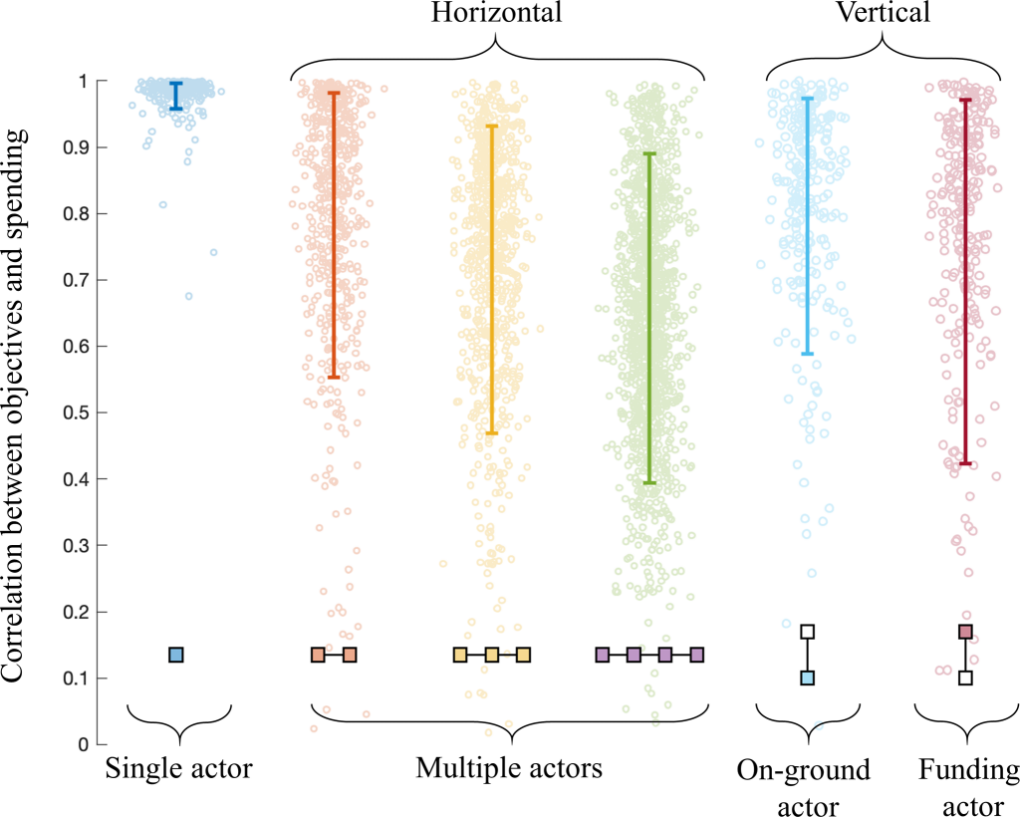
Alignment between the objectives and resource allocation in multi-actor conservation systems (measured by Pearson's correlation coefficient, ***r***), based on strategic prioritizations in 250 simulated systems. The boxes depict the number of hypothetical actors and their relationship to each other. The dots on the plot represent the outcomes for individual actors, and the error bars represent the 95% confidence interval. In single-actor systems, there is a high degree of alignment between the actor's objectives and resource allocation (the first column). However, when multiple actors are behaving reactively, optimal resource allocation decisions result in misalignment (columns 2–6). This misalignment is present in both horizontally (columns 2–4) and vertically (columns 5–6) structured systems and tends to increase with the number of actors (i.e., the increasing number of boxes in the figure). For vertical systems, misalignment differs between the on-ground actors (column 5) and the funding body (column 6).

In the absence of multiple actors, our model predicts that the application of prioritization tools will result in a close correlation between an actor's objectives and their spending (figure 4). That is, our model predicts the same alignment between objectives and spending that the conservation literature expects to see when prioritization tools are being applied.

By contrast, when prioritization tools are applied in multi-actor systems with horizontal structure, spending no longer aligns with an actor's objectives (figure 4). This is because actors are making allowances for decisions made by the other actors in the system. As the number of actors increases, the alignment between spending and objectives decreases further (figure 4).

If the conservation sector contains vertical structure, then our models predict the same low alignment between spending and objectives—for both the on-ground actor and the funder (figure 4). Although the funder has the advantage of acting first in the Stackelberg game, their spending decisions are less aligned with their objectives than for the on-ground actor. This misalignment is a reflection of their more powerful role in the interaction. The funding actor knows that the on-ground actor will allocate resources to features that both organizations consider high value. The funder is therefore free to reduce their allocation to these features, creating a misalignment.

### Cooperative behavior reduces poor conservation outcomes for individual actors

**Figure 5.**
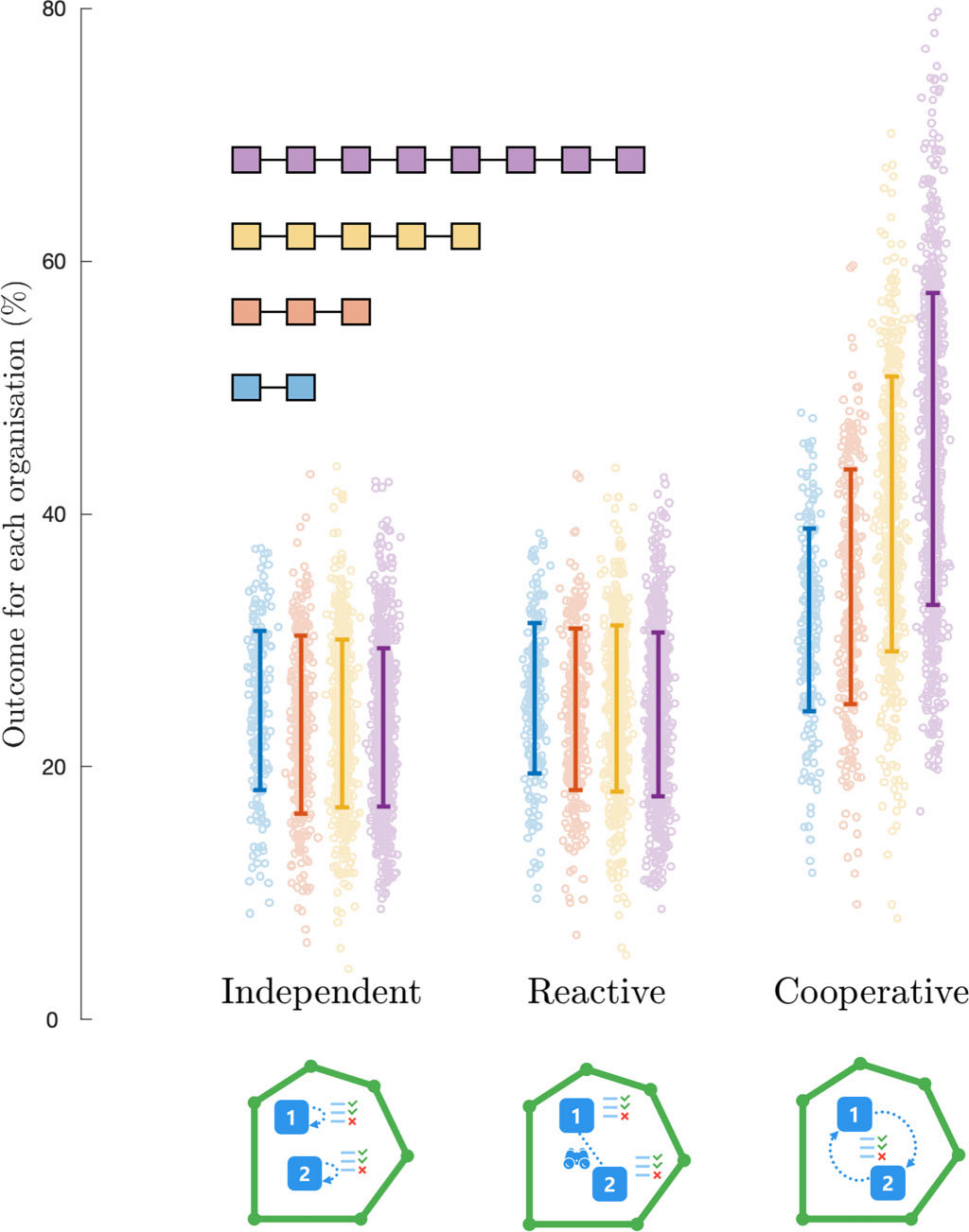
The percentage of the conservation goals achieved by each actor, because of their independent, reactive, or cooperative behavior, based on strategic prioritizations in 250 simulated systems. In this case, an outcome of 100% could be achieved if an organization's funding was unconstrained. The boxes represent the number of hypothetical actors in a system as they appear in each column, the dots on the plot represent individual actors, and the error bars represent the 95% confidence interval. When actors prioritize actions independently of or reactively to each other, the percentage of conservation goals achieved tends to be low, and this appears to hold regardless of the number of actors in the system. However, when actors work cooperatively with each other to prioritize actions, then the percentage of conservation goals achieved generally tends to be higher, especially as the number of actors increases in the system.

We find that the percentage of an actor's conservation goals achieved from prioritization exercises is higher for actors who cooperate than for actors who act independently or reactively (figure 5). In figure 5, the same total amount of funding is shared between an increasing number of actors, who behave either independently, reactively, or cooperatively with each other.

Our models further support the literature consensus that conservation outcomes are worse for individual actors when they behave independently or reactively than for those that act cooperatively (figure 5; Bode et al. 2011, Gordon et al. 2013, Kark et al. 2015). Regardless of the number of actors, cooperative prioritizations achieve much better conservation outcomes than either independent or reactive interaction behavior. Independent actions result in poor outcomes for actors because conservation features that are attractive to many actors receive too much funding at the detriment of other features. Similarly, when individual actors are reactive—when they recognize and strategically react to the resource flow and actions of other actors but do not actively cooperate with other actors—then their percentage of conservation goals achieved are comparable to independent action. That is, behaving reactively toward other actors in the sector is no better than ignoring them (assuming all organizations are doing the same thing).

As we might expect, the presence of larger numbers of actors degrades the outcomes of independent and reactive behavior. If they are not cooperating, then more actors simply represent more opportunities for competition and conflict. However, the effect is quite small, even for relatively large increases in the size of the sector (e.g., from two actors to eight actors; figure 5). Larger sectors increase the benefits of cooperative behavior. Interestingly, this is because cooperative behavior delivers better outcomes, not because the outcomes of reactive and independent behavior get worse.

## Challenges for effective priority setting in multi-actor systems

Conservation theory has begun to acknowledge the multi-actor nature of the conservation sector (Albers et al. 2008, Bode et al. 2011, White et al. 2012, Gordon et al. 2013), but this theoretical realization has not yet altered the tools that conservation managers generally use to plan their decisions. Most conservation prioritization tools begin with the assumption that a single actor (the actor using the prioritization tool) has fiat power to change all conservation resource allocations. The existence of other actors—let alone their decisions—is not considered (Joseph et al. 2009, Gerber 2016, Gerber et al. 2018).

Box 1. **Example game of simple marginal swap.**

A landscape contains four habitat patches, which each contain two threatened species. The abundance of each species—a proxy for the benefit achieved by their protection—is contained in an ordered pair. The habitat patches are these:

\begin{equation*}{h}_1 = \left( {0,\ 10} \right)\end{equation*}

\begin{equation*}{h}_2 = \left( {10,\ 0} \right)\end{equation*}

\begin{equation*}{h}_3 = \left( {7,\ 8} \right)\end{equation*}

\begin{equation*}{h}_4 = \left( {8,\ 7} \right)\end{equation*}

Two taxon-specific conservation organizations operate in the landscape. The first is only interested in the abundance of the first species (the first element in each ordered pair); the second is only interested in the abundance of the second species. Each organization can afford to protect only one habitat patch, but they receive a benefit if a patch is protected by either organization.

Left to their own devices, organization 1 would pursue the protection of patch 2, and organization 2 would protect patch 1. However, their individual benefits and their summed benefits would be maximized by the protection of patches 3 and 4. These two patches are not superlatively valuable for either organization when considered individually. However, together, they can protect a larger abundance of each species than any other combination of patches.

We can structure the decision as a classic discrete game, where the relevant payoff matrix is this:

**Table:** 

	Organization 1 decision		
Organization 2 decision		Protect ${h}_2$	Protect ${h}_4$
	Protect ${h}_1$	[10, 10]	[8, 17]
	Protect ${h}_3$	[17, 8]	[15, 15]

In a classic prisoner's dilemma, the Nash equilibrium occurs when patches 1 and 2 are protected, despite the social-good benefit being maximized when patches 3 and 4 are also protected.

New conservation prioritization tools are therefore needed that allow for more than one actor to have agency for changing conservation outcomes. However, these will be socially, economically, and computationally challenging to implement (Epanchin-Niell et al. 2010, Gordon et al. 2013, Bodin 2017, Sierra-Altamiranda et al. 2020). These transaction costs are diverse, including the costs of gathering information, the costs of bargaining over benefits or costs, and the costs of monitoring and enforcing the resulting agreements, among others (Wondolleck and Yaffee 2000, McCann et al. 2005, Marshall 2013, Lubell et al. 2017). Transaction costs are extensive in systematic conservation planning where collaboration is key (McDonald 2009, Bode et al. 2011) and more generally in collaborative partnerships in complex environmental institutional systems (Lubell 2015). Therefore, options that minimize complexity, minimize transaction costs, and maximize trust are essential components for effective cooperative prioritizations in the interim (Guo and Acar 2005, Perrault et al. 2011, Kark et al. 2015, Bodin et al. 2020).

## Toward effective conservation prioritizations in multi-actor systems

Collective decision-making through deliberation is essential for avoiding poor outcomes for individual actors, and this deliberation may involve repeated and reciprocal commitments to build trust. One way to do this is through multiple interactions that produce small wins that build trust, reputations, infrastructure, and resources, which can enable bigger wins in future (Ostrom and Walker 2003, Termeer and Dewulf 2019, Lubell and Morrison 2021). Approaches will consequently need to be developed that allow small, sequential collaborative steps where actors can incrementally assess areas of agreement in their prioritizations (Bode et al. 2011, Gordon et al. 2013, Kark et al. 2015, Lubell and Morrison 2021).

One example of an incremental decision-making tool for multiple actors is the C-plan conservation planning system (Pressey et al. 2009). C-plan software was developed in the 1990s to support negotiations on regional forest agreements and conservation planning in New South Wales, Australia. The software allowed users to interactively and visually compare the consequences of their independent actions in a facilitated group setting. Although it did not attempt to undertake a multi-actor prioritization, C-plan could reveal that small allowances by the actors may deliver mutually beneficial decisions (Finkel 1998). Similarly, simulations of conservation actors who purchased land parcels cooperatively by forgoing their top preferences tended to have higher overall land protection than those who purchased land parcels independently (Bode et al. 2011). We believe that prioritization theory could learn from these findings, and we propose a practical step-by-step process for actors to work toward areas of small wins through a series of marginal and mutually beneficial allowances, or marginal swaps, as part of the conservation prioritization process (Bode et al. 2011, Colyvan et al. 2011).

**Figure 6.**
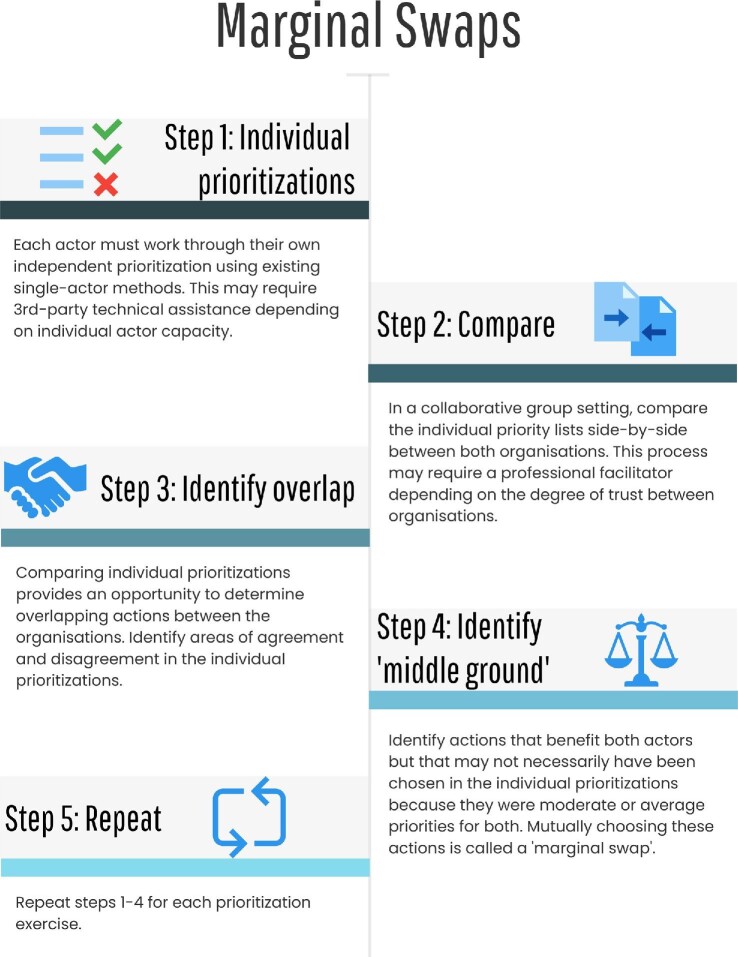
Steps for identifying marginal swaps for effective prioritizations in multi-actor conservation systems. The first step in a marginal swap exercise is for the participating actors to conduct individual prioritizations using standard methods (step 1). Then, the actors compare their individual prioritizations side by side in a collaborative environment (step 2) and identify overlapping priorities (i.e., areas of agreement or disagreement) between the actors (step 3). From these overlapping priorities, the actors then identify features that are mutually valued, which are likely to not be highly ranked in each actor's prioritization and therefore represents a swapping of their own top priorities for intermediate priorities that are mutually beneficial (step 4). These steps can be repeated for each prioritization exercise (step 5).

We suggest that conservation prioritization theory engage with the challenges of multi-actor conservation by building on existing methods incrementally, rather than with radical new tools. Our proposed process, which is based on marginal swaps, consists of four key steps (illustrated in figure 6). The process starts with each actor working through their own single-actor prioritizations, using existing tools and their own objectives (figure 6, step 1). This step will determine priorities that are consistent with each actor's objectives. The next step is to compare individual actor's priorities in a collaborative environment (figure 6, step 2; Frank and Sarkar 2010). This is useful to identify overlapping actions, including actions that are mutually beneficial (figure 6, step 3), which may require multiple meetings for deliberation and negotiation around conflicting priorities (Pressey et al. 2009, Frank and Sarkar 2010). Some of the mutually beneficial actions identified during this process may not have been ranked as a top priority by any individual prioritization, because they do not offer superlative results for any actor. Swapping their own priorities for intermediate priority actions represent marginal swaps (figure 6, step 4). In box 1, we provide a simple example illustrating this process, with two actors making decisions to protect species abundance within habitat patches. The best overall outcome across the entire conservation system is achieved when the actors forego their preferred species’ habitat patches to jointly conserve other habitat patches that produce a larger abundance of species.

## Conclusion

Conservation landscapes generally contain multiple actors, and each can choose to ignore, recognize, or cooperate with the others. In the present article, we illustrate that, for the first two choices—ignoring or reacting to each other's decisions—actors will likely achieve fewer conservation goals. Moreover, we suggest that actors can harness the mutual benefits of cooperation if they switch priorities in small collaborative steps via marginal swaps. Marginal swaps do not require new tools; rather, they require each actor to use standard prioritization methods as if they were prioritizing for their own objectives (i.e., single-actor methods) and then compare the outcomes of the two independent prioritizations in a stepwise collaborative manner.

There is little doubt that cooperative plans will require actors to swap their individual top-priority actions for intermediate, mutually beneficial actions. Through this process, marginal swaps can drive an apparent misalignment between actors’ objectives and resource allocation. As such, although misalignment can reflect bad decision-making and poor outcomes for individual actors, it can also be a byproduct of cooperative planning and cannot be a metric used to measure the efficiency of conservation spending.

Although marginal swaps represent a step in the right direction for mutually beneficial outcomes in multi-actor conservation systems, there is much opportunity for enhancing conservation prioritization methods that explicitly capture the complexity of multi-actor systems. A future direction may be to investigate the costs and benefits of conducting a more complex optimization that factors in cooperative priority settings versus classic independent prioritization techniques. New methods may also incorporate the decisions made by actors outside of the conservation system, which adversely influence the utility of individual conservation actors (e.g., actors focused on agriculture, resource extraction, recreation). The addition of these actors would require prioritization approaches that account for divergent and competing objectives. Developing a better understanding of the trade-offs between complex prioritizations and more mainstream methods is an important future research agenda for deciding whether and when it is worth investing in methods that explicitly incorporate the intricacies of multiple actors. Until such a method is widespread and repeatable, decision-makers must jointly make small steps toward cooperation to reduce poor outcomes for the values they are trying to protect.

## Supplementary Material

biad046_Supplemental_File

## Author Biographical

Christopher J. O'Bryan (c.obryan@uq.edu.au) is a postdoctoral research fellow, Jonathan R. Rhodes is a professor, Nisansala Abeysinghe Mudiyanselage is a PhD student, and Eve McDonald-Madden is a professor in the School of Earth and Environmental Sciences and the Centre for Biodiversity and Conservation Science at The University of Queensland, in Brisbane, Queensland, Australia. Olusegun O. Osunkoya is a principal research scientist and Moya Calvert is a senior biosecurity officer for the Invasive Plant and Animal Science Unit, in the Department of Agriculture and Fisheries at Biosecurity Queensland, in Brisbane, Queensland, Australia. Geoff Lundie-Jenkins is a director for Wildlife and Threatened Species Operations, in the Department of Environment and Science at Queensland Parks and Wildlife, in Toowoomba, Queensland, Australia. Travis Sydes is a manager for the Far North Queensland Regional Organisation of Councils, in Cairns, Queensland, Australia. Michael Bode is a professor in the School of Mathematical Sciences at Queensland University of Technology, in Brisbane, Queensland, Australia.
