# Supplementary material for: Setting conservation priorities in multi-actor systems
Source: Bioscience. 2023 Jul 19;73(7):522–32. doi: 10.1093/biosci/biad046 (PMC11616722; doi:10.1093/biosci/biad046)
Supplement: biad046_Supplemental_File [file biad046_supplemental_file.docx]

**Supporting Information for “Setting Conservation Priorities in Multi-Actor Systems”**

Christopher J. O’Bryan^1,2*^, Jonathan R. Rhodes^1,2^, Olusegun O. Osunkoya^3^, Geoff Lundie-Jenkins^4^, Nisansala Abeysinghe Mudiyanselage^1,2^, Travis Sydes^5^, Moya Calvert^3^, Eve McDonald-Madden^1,2†^, Michael Bode^6†^

^1^School of Earth and Environmental Sciences, The University of Queensland, Brisbane QLD 4072, Australia

^2^Centre for Biodiversity and Conservation Science, The University of Queensland, Brisbane, QLD 4072, Australia

^3^Invasive Plant and Animal Science Unit, Department of Agriculture and Fisheries, Biosecurity Queensland, Ecosciences Precinct, Brisbane, QLD 4102, Australia

^4^Wildlife and Threatened Species Operations, Department of Environment and Science, Queensland Parks and Wildlife, Toowoomba, QLD 4350, Australia

^5^Far North Queensland Regional Organisation of Councils, Cairns, QLD, 4870 Australia

^6^School of Mathematical Sciences, Queensland University of Technology, Brisbane, QLD 4000, Australia

*Correspondence: [c.obryan@uq.edu.au](mailto:c.obryan@uq.edu.au)

^†^Authors should be considered joint senior author

**Example iterative solution of a Nash equilibrium**

Consider a landscape where two organisations have unit budgets and seek to conserve two conservation features: $r\in\left\{ 1,2 \right\}$, $f\in\left\{ 1,2 \right\}$. The cost of protecting each feature is $c_{f}=\left\{ 0.8, 0.6 \right\}$. The first conservation organisation attributes values to the features of $\alpha_{1f}=\{0.6 0.4\}$. The second conservation organisation attributes similar relative values to the features, but with slightly more emphasis on the first feature: $\alpha_{2f}=\{0.7 0.3\}$.

Both organisations initially allocate funding to the two features in a proportion equal to their relative values. Thus, organisation 1 puts 60% of its funding into feature 1, and the remaining 40% into feature 2. Organisation 2 puts 70% into feature 1.

Following the iterative gradient search algorithm, we allow each organisation in turn to re-allocate a small proportion of its funding from either feature 1 to feature 2, or the other way around. The changed allocation is accepted if it increases the utility of the organisation that made the change (the effect of the change on the other organisation, which will often be negative, is not considered).

Figure S1 shows the resulting evolution of the spending patterns of the two organisations. Because the spending on feature 2 is simply the complement of the spending on feature 1 (i.e., because $\beta_{r1}=1-\beta_{r2}$), we can visualise the two strategies on a pair of 2D axes, with the utility of the two organisations shown in colour on two panels.


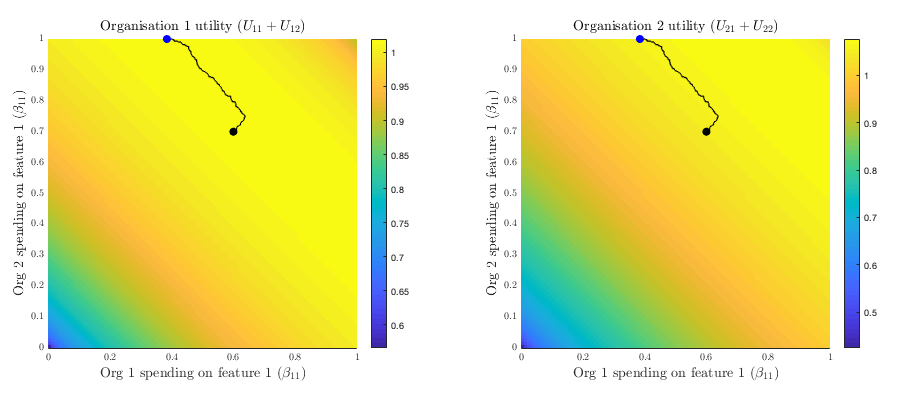


*Figure S1: Identification of a Nash equilibrium spending policy for two organisations in a single landscape. The initial allocation is in proportion (i.e., perfectly related) with the relative value assigned by the organisations to the two features. However, the organisations choose unilateral allocation changes that improve their marginal utility, and in doing so organisation 1 shifts 20% of its funding from feature 1 to feature 2, and organisation 2 shifts 30% of its funding from feature 2 to feature 1. The result is (1) a break in the relationship between an organisations’ values and its spending, and (2) an improvement in the total utility for both organisations, particularly for organisation 2.*
